# The Continuum Between Temperament and Mental Illness as Dynamical Phases and Transitions

**DOI:** 10.3389/fpsyt.2020.614982

**Published:** 2021-01-18

**Authors:** William Sulis

**Affiliations:** Collective Intelligence Laboratory, Department of Psychiatry and Behavioural Neuroscience, McMaster University, Hamilton, ON, Canada

**Keywords:** temperament, mental illness, dynamical phase, phase transitions, critical markers

## Abstract

The full range of biopsychosocial complexity is mind-boggling, spanning a vast range of spatiotemporal scales with complicated vertical, horizontal, and diagonal feedback interactions between contributing systems. It is unlikely that such complexity can be dealt with by a single model. One approach is to focus on a narrower range of phenomena which involve fewer systems but still cover the range of spatiotemporal scales. The suggestion is to focus on the relationship between temperament in healthy individuals and mental illness, which have been conjectured to lie along a continuum of neurobehavioral regulation involving neurochemical regulatory systems (e.g., monoamine and acetylcholine, opiate receptors, neuropeptides, oxytocin), and cortical regulatory systems (e.g., prefrontal, limbic). Temperament and mental illness are quintessentially dynamical phenomena, and need to be addressed in dynamical terms. A meteorological metaphor suggests similarities between temperament and chronic mental illness and climate, between individual behaviors and weather, and acute mental illness and frontal weather events. The transition from normative temperament to chronic mental illness is analogous to climate change. This leads to the conjecture that temperament and chronic mental illness describe distinct, high level, dynamical phases. This suggests approaching biopsychosocial complexity through the study of dynamical phases, their order and control parameters, and their phase transitions. Unlike transitions in physical systems, these biopsychosocial phase transitions involve information and semiotics. The application of complex adaptive dynamical systems theory has led to a host of markers including geometrical markers (periodicity, intermittency, recurrence, chaos) and analytical markers such as fluctuation spectroscopy, scaling, entropy, recurrence time. Clinically accessible biomarkers, in particular heart rate variability and activity markers have been suggested to distinguish these dynamical phases and to signal the presence of transitional states. A particular formal model of these dynamical phases will be presented based upon the process algebra, which has been used to model information flow in complex systems. In particular it describes the dual influences of energy and information on the dynamics of complex systems. The process algebra model is well-suited for dealing with the particular dynamical features of the continuum, which include transience, contextuality, and emergence. These dynamical phases will be described using the process algebra model and implications for clinical practice will be discussed.

## Introduction

The concept of a conjoining of biological, psychological, and sociological domains into a single framework for understanding the origins, developmental courses, and established trajectories of various forms of mental illness has been around for several decades. It provided the fundamental framework for conceptualizing and managing mental illness during the author's residency training. Its rather simplistic application in those days belied the extraordinary complexity involved in the interactions between these three domains. Nowadays the focus has shifted toward the notion of biopsychosocial complexity highlighting the specific contribution that the presence of *complexity* brings to understanding mental illness. Complex systems theory (1–3) as a separate domain of inquiry emphasizes the roles of non-linearity, chaos, intrinsic randomness, self-organization, emergence, criticality, self-organized criticality, fluctuations, power laws, non-normal probability distributions, among many others. These stand in stark contrast to simple, linear systems theory (4) where the emphasis is on stability, equilibrium, normal probability, noise.

It is odd that the DSM describes states of mental illness without reference to the transitions into and out of illness. In the DSM, a person is either normal, or ill. There is no reference to becoming ill, or becoming well. This absence may have contributed to the conflict that the author noted between psychiatrists and general practitioners during his residency training. General practitioners would often send patients to the outpatient psychiatry clinic with a diagnosis of depression, only to have the patient rejected by the clinic, and then re-appearing some weeks or months later to be diagnosed with depression. In retrospect it was apparent that the general practitioner was observing a patient in evolution, transitioning from normality into depression. They were seeking early intervention, which never happened because the only reference point for the psychiatrist was a well-established depressive state.

The physical sciences have been dominated by the conservation laws, particularly conservation of energy, which became viewed as the dominant currency of the natural world. In recent years there has been increasing recognition of the role that information plays in the dynamics of physical systems (5). Within the neurosciences, research into information flow within neural systems (6) has demonstrated the presence of additional dynamical features which are important in understanding biopsychosocial complexity. These include transience, fungibility, generativity, contextuality, and deep emergence. These pose deep problems for the modeling of biopsychosocial interactions.

Attempts to understand mental illness have all too often been directed at a single ontological level, with the focus shifting from biological (Hippocrates) to psychological (Freud, Jung, Adler) to sociological (Durkheim) back to biological (serotonin-noradrenaline) to psychological (cognitive behavior theory) to sociological (transactional analysis) and so on. There have been far fewer attempts to develop a truly integrative, multi-level, multi-scale, multi-system theory, whether of normative function or of mental illness. The focus on biopsychosocial complexity provides an important starting point toward such theory building.

Why does this matter? Truth be told, psychiatry has made very little real progress over the past decades. Newer antidepressants (apart from the promise of Ketamine) are simply variants on the same old paradigm (stimulate noradrenaline or serotonin) and psychotherapy has become replaced by psychoeducation, teaching people a collection of useful cognitive tricks to correct so-called biases or distortions, with no real meaning, understanding or healing attached to it. Psychoanalysis appeared to be all heart with a flawed theory, while modern treatments appear to be mostly technology with an empty heart. The study of biopsychosocial complexity embraces the totality of the human experience, seeking integration, understanding and ultimately a path to healing. It takes the proverbial blind men, having examined the elephant, and brings them together to create a synthesis of perceptions and conceptions and to integrate them into a whole which ultimately may lead to healing.

The sheer complexity of human experience, however, suggests the need to focus upon a smaller range of phenomena and systems in an effort to make the process of theory building more tractable without sacrificing the inherent complexity. The goal of this paper is to motivate one particular focus for study, that of the temperament-mental illness continuum. The rationale for this suggestion will be provided in the next section, followed by a brief discussion of some experimental, theoretical and clinical forays into this subject. A particular model, the Process Algebra model, will be presented and one particular model of the continuum as a space of higher order dynamical phases and transitions will be described.

## The Temperament-Mental Illness Continuum

Several authors have suggested that temperament and mental illness can be viewed as lying along some form of continuum (7–14). This idea can be traced back to Hippocrates who suggested that temperament and mental illness can be understood as being caused by different levels of four humors within the body. Modern views of this continuum also involve neurochemistry and neuroanatomy (both structural and functional) but more importantly they have moved beyond simple quantitative or dimensional factors to consider the role of dynamics and its structure and symmetries.

Temperament refers to biologically based, consistent, enduring, *patterns* of normative behavior. Mental illness, on the other hand, refers to consistent, enduring, dysfunctional or pathological *patterns* of behavior. In these definitions the emphasis is on *temporal patterns*. Both temperament and mental illness describe distinct long term patterns of behavior. Here, behavior is to be understood in a broad sense as referring to temporally organized sequences of motor acts, or of cognitive or affective states, which appear in specific contexts (which may be internal or external) and to which can be attributed some semiotic, functional or ecological value. Temperament and mental illness are not defined by single (or even a few) individual behavioral acts; rather, it is the long term temporal patterning of a range of acts, together with their contextual linkages, which is important. This is strikingly illustrated by the seminal work of Gottschalk et al. (15), who showed that subjects with bipolar disorder and control subjects were distinguished, not merely by the intensity of reported moods but by the temporal patterning of mood shifts. The mood changes of control subjects over the course of a year proved similar to white noise, while those of bipolar subjects showed a wide diversity of temporal patterning, sometimes noisy, sometimes ordered, sometimes persistent, sometimes intense, sometimes normative; recurrent, but not truly periodic.

Neither temperament nor mental illness has been seriously studied with this dynamical patterning in mind. For the most part, both have been studied through self-report questionnaires which ask subjects to reflect upon and assess the average occurrence of some behavior or characteristic of behavior. The time interval might be the life span, or the interval between therapy sessions. The nuances associated with these behaviors are lost. In spite of this it has been recognized that temperament and mental illness share a number of features which support the notion that they lie along some continuum.

First of all, temperament and many chronic mental illnesses, particularly the affective disorders, share the same underlying neurobehavioral regulatory systems (NBRS). These include neurochemical behavioral regulatory systems (NCRS) (such as monoamine, acetylcholine, GABA, Glutamate, opiate receptor, neuropeptide, oxytocin, and hormome systems) and cortical regulatory systems (CRS) (such as prefrontal, frontal, and limbic systems) (7, 8, 16–20). These diverse regulatory systems are organized across multiple spatiotemporal scales, possess complex feedback relationships, and are capable of expressing a remarkable diversity of behavioral patterns.

Second, many DSM symptoms of illness bear a similarity to certain temperament traits, particularly those comprising the Functional Ensemble of Temperament (FET) model (12, 14, 21–26). Most models of temperament have been based upon the lexical approach, whose source material consists of verbal descriptors of behaviors (27, 28). Most of these descriptors relate to emotionality and sociability. The FET, on the other hand, is based upon research into neurobiology, neurophysiology, psychophysics and neuropsychology and divided traits along two lines: the domain of activity (intellectual, physical, social) and the dynamics of activity (endurance, variability, orientation). This results in 9 traits which are supplemented by 3 emotionality amplifier traits (neuroticism, self-confidence, impulsivity). Every trait within the FET model is associated, not with a single neurotransmitter system, but with a specific, distinct ensemble of NCRS (7).

Temperament traits linked to NCRS include impulsivity, sensation seeking, neuroticism, endurance, plasticity, and sociability (7, 8). DSM symptoms linked to these NCRS include: psychomotor retardation [shown to be regulated mostly by dopaminergic systems (25), modulated by mu-opioid and delta-opioid receptor systems], fatigue [linked to serotonin systems (26) which are linked to depression (29–31) and to endurance (26, 31)], impulsivity [linked to interactions between delta opioid, mu-opioid and dopamine systems (32)], dysphoria [linked to mu-opioid receptor systems (33, 34)], anxiety [linked to impairment in the regulation of kappa opioid receptor systems by mu opioid receptor systems, arising due to effects on GABA (35–37), noradrenaline and the HPA axis (38)].

Third, it has been conjectured that if temperament and mental illness lie along a continuum, and if the temperament model is sensitive to changes in activity in NCRS, then, in the presence of at least some forms of mental illness, there should be differential impacts on temperament traits in the presence of these illnesses. Standard temperament models such as Big Five (39), positive-negative affect (40) or TCI (18) have shown no or at best modest differential effects and appear unable to distinguish between major depression and generalized anxiety disorder. Affective temperament models such as Akiskal's Affective Temperament Scale (41) or the AFECT model (42), are promising in so far as mood disorders are concerned but have not yet been shown to possess differential sensitivity in the presence of a broad range of illnesses. On the other hand, the FET has shown differential effects in the presence of several categories of mental illness and in particular, differentiates between major depression, generalized anxiety disorder, comorbid depression and anxiety, and several personality and psychotic disorders (12, 21–24). This result showing differential effects in the presence of illness makes the FET model the most promising model currently for exploring the temperament-illness continuum and the value of basing a model on neurophysiological evidence rather than lexical descriptors (7, 8, 13, 14, 21–24).

It is important point to note that temperament is normative, even in the presence of mental illness. Temperament scores may shift in the presence of mental illness but seldom extremize. A simplistic dimensional model of the temperament-illness continuum is not supported. Nevertheless, there do indeed appear to be linkages between temperament and mental illness, but a more nuanced approach appears to be required. The main conjecture of this paper is that an understanding of the temperament-mental illness continuum is to be found in the dynamics of NCRS and NBRS. An understanding of the continuum could play an important role in individualized diagnosis and treatment planning (as temperament is a core source of individual differences), and an understanding of the dynamics involved could pave the way for new biomarkers for states of illness but also for transitions between such states, which is important for treatment monitoring.

## Dynamical Systems Theory in Psychiatry

Like its sister discipline psychology, psychiatry is all about behavior, in particular behavior that occurs because of damage to or dysfunction of the nervous system. Behavior refers to sets of ecologically homologous behavioral acts. A behavioral act is a finite duration sequence of states, which may be motoric, cognitive or affective, carried out by an organism, usually on a short time scale of seconds to minutes, which express some ecologically valid biological, vocational or social function in a specified context. Behavior, by definition, is dynamic, as it involves changes over time. Behaviors are generally organized into sequences, or temporal patterns, which take place over minutes to hours, are correlated with environmental stimuli and possess a functional character. These sequences of behaviors (termed activities) make up a larger tapestry of patterns which persist over weeks, months or years. These long term patterns comprise temperament and mental illness.

Dynamical systems theory is the formal study of systems that change in time. The study of dynamical systems has been primarily a subject of mathematics and physics, but its adoption in fields such as psychology and psychiatry is long overdue. Nevertheless, a small but growing number of researchers have been attempting to apply the methods of dynamical systems theory at both theoretical and clinical levels (43–55).

Dynamical systems theory seeks out symmetries and universal characteristics of dynamics in general. The classification of trajectories forms the basis for the geometrical approach to dynamical systems. Trajectories may be described in terms of their final destination, their attractors. The most common attractors are (1) fixed point, (2) periodic, (3) quasiperiodic, (4) strange (chaotic). Trajectories which do not trend toward an attractor may be divergent, transient, recurrent or ergodic (56). There has been a great deal of interest in characterizing behaviors which occur at the boundaries between differing patterns of behavior, in particular that between laminar and chaotic patterns. One of the most studied transitional patterns is that of intermittency (alternations of periodic and chaotic patterns) (57–59), of which several distinct types have so far been described (I,II,II, on-off, eyelet, ring) (60, 61). Intermittency has been studied mostly for a single dynamics in which certain trajectories bounce between regions of laminar dynamics and chaotic dynamics. Ansmann, Lehnertz and Feudel (62) studied such self-induced switchings on a complex network, showing spontaneous transitions between low-amplitude oscillations, non-linear waves and extreme events, which appeared random, but were due to the presence of a chaotic saddle. Both short and long range connections played a role but short dominated. A similar chaotic saddle has been observed underlying intermittency in wave motion in fluids (60). Interestingly, it is possible to have intermittent intermittency (alternation of two different types of intermittency) (63, 64). Intermittency is widespread and has been observed in EEG signals (61, 64). Some measures of intermittency such as the reinjection probability density has been developed or short time series, and thus possibly suitable for clinical applications (59).

The analytical approach has been effectively applied to the analysis of time series data (65–67). Analytical tools, currently in widespread use and suitable for studying behavioral data include: (1) Fluctuation spectroscopy, (2) Fluctuation distributions (these tend to be power law rather than Gaussian), (3) Lempel-Ziv complexity, (4) Hurst exponent, (5) Lyapunov exponent, (6) Shannon and Renyi entropy, (8) Ap entropy, (9) Mutual information, (10) Recurrence and dwell times, (11) Recurrence plots, (12) Orbital Decomposition, (13) Correlation, Hausdorff and Box dimensions, (14) Scaling, (15) Order parameters. The list continues to grow as techniques advance.

There have been several attempts at formal modeling relevant to psychiatry [see also (14) for a more detailed survey], particularly related to serotonin and dopamine dynamics (68–75). The earliest work on the continuum between temperament and mental illness is that of Mandell and Selz (76), who proposed perhaps the first formal model of the temperament-mental illness continuum, though strictly speaking they focused on personality disorders (borderline and obsessive compulsive) which straddle the gray middle ground. They focused on dynamical patterning in behavior, using an attractor based model, finding that spatial entropy and largest Lyapunov exponent appeared to distinguish these disorders. Remarkably little subsequent work has been carried out though a few papers have explored models of personality (77), temperament (78). A great deal of attention has been paid to mood disorders (79–95). Other topics studied include psychosis and cocaine addiction (96, 97).

Time series methods have been used widely in both basic science (98) and are gaining importance in clinical research where they have been applied to the study of bipolar disorder (99–103), schizophrenia (104), obsessive compulsive disorder (98), generalized anxiety disorder (105–110) among others. Data for these time series are taken from a wide variety of sources including heart rate, gait monitors and most commonly, personal ratings of mood.

An important application of these ideas has been to the theory and monitoring of treatment progress. Two distinct theoretical approaches have been a continuous time model based on ideas from synergetics (111) and an empirically motivated discrete time model (112). Patterns of change in psychotherapy have been examined from a dynamical systems perspective (113). Time series have been used to study mood fluctuations, which is important for understanding the course of treatment (99–101, 114–116).

In the case of bipolar disorder, formal models have tended toward cartoon stereotypes, either exhibiting too much regularity or periodicity (like a sine wave) (81, 82) or too much variability (83) resembling noise. For example, a complex systems model positing winnerless competition between two recurrent maps, one for GABA and one for Glutamate reproduced several features seen in bipolar patients, particularly intermittency (84) but they appear too noise-like, and none have reproduced the prolonged dwell times, recurrence and persistence, intermittency, slow wave and long wave patterning observed in Gottschalk et al.'s bipolar subjects (15).

Two interesting papers touch on matters relevant to the temperament-mental illness continuum and the FET model. A network analysis was used to assess the centrality of DSM vs. non-DSM symptoms and somewhat surprisingly found little difference between them (92). A causal network analysis examined the relationship between symptoms of depression and anxiety, finding that symptoms were more closely related within each disorder rather than between disorders (93). This is in keeping with the FET model which showed that major depression, while affecting 9 temperament traits, nevertheless had a greater effect on the physical traits, whereas generalized anxiety disorder, which affected 5 traits, had a greater impact on the social traits. In addition the authors found that “low energy” had high centrality for major depression, again consistent with the FET finding of significantly lower physical endurance and tempo in the presence of major depression.

Additional support for the FET model comes from research into various aspects of movement such as gait (117), and activity (118, 119). Radovanovic et al. (117) found that subjects with major depression had slower speed and decreased gait variability compared to controls, consistent with the FET finding of decreased motor tempo among subjects with major depression. Kim et al. showed that in the presence of major depression, patients exhibit reduced mean activity levels punctuated by occasional burst of activity (increased intermittency) (119).

Prior applications of dynamical systems methods in psychiatry have mostly focused on the use of attractors to model various psychiatric states but mostly as metaphors (48–50). Complex systems theory has been used to explore the idea of depression as a “stuck state” of emotional processing (85). Stochastic cellular automata have been used to model the interaction between symptoms and mood states and their transitions (86). These are essentially qualitative models at the symptom level.

Transitions into and out of clinical states have traditionally been studied through the lens of epidemiology, looking for correlations with rather coarse risk factors such as age, sex, family history, premorbid history, and genetics. Such studies have examined transitions into depressive or anxious states (120–127) or among bipolar states (113, 128–131). One formal study examined the pattern of survival rates for patients transitioning out of an episode of major depression, modeling it as a diffusive Ornstein-Uhlenbeck stochastic process (132). The transition from pathological to normal mood was treated like a diffusion or infective process, one mood state gradually transitioning to the other.

Interest has been growing in recent years, particularly within the psychotherapy community, in identifying biomarkers or physiological signals/signs which could be used to track progress in psychotherapy. Such knowledge is vitally important to the clinician so that appropriate interventions can be undertaken to either ameliorate the risk or facilitate on-going change. Gait (117–119), sleep, (133) and functional connectivity (134) have been proposed as possible biomarkers. However, the choice of effective biomarkers depends upon our knowledge of the nature of the states and of their transitions, to which we now turn.

## Dynamical Phases, Phase Transitions, and The Continuum

Everyone is familiar with the fact that water, at different temperatures, possesses distinct phases which are distinguished by a number of intrinsic characteristics such as density, conductivity, tensile strength, specific heat, compressibility, viscosity, and so on. These characteristics persist over a range of contexts described by (controllable) variables such as temperature, volume, pressure etc. Likewise, particular characteristics of behavior persist across a range of environmental, social and occupational contexts. These persistent characteristics constitute the behavioral equivalent of a phase of matter. When normative, they are referred to as temperament. When maladaptive (pathological) they become expressions of chronic mental illness. Physicists have long been interested in understanding not only the structure of these distinct phases but also the transitions which take place between them as the controllable parameters are varied (135). Likewise, therapists have been interested in the transitions between normative temperament and pathological illness and vice versa. Physical phase transitions have turned out to exhibit some remarkable properties—universality being perhaps the most striking. It has been found that for many transitions, the functional form of the relationship between the internal characteristic of interest, say specific heat, and the controllable parameters, is universal in the region of the controllable parameters surrounding the point at which the transition occurs (136, 137). Moreover, in the region surrounding a transition, measurements of various attributes may fluctuate, and these fluctuations often follow particular distributions, in particular power law distributions. This has allowed physicists to identify several markers to indicate when a transition is at hand (138).

A comparable notion of phase and phase transition in neurobiology and psychiatry is much more challenging. Matter phases are static. However, time and dynamics are fundamental to the concept of behavior, requiring a concept of dynamical phase and dynamical phase transition. Behavioral systems are by their very nature, open systems, and contextuality also plays a fundamental role. Matter phases are stable, and behaviors too are generally stable, at least relative to the specific functionality being expressed by the behavior. A behavioral response should remain more or less the same over a wide range of conditions. It does an organism no good if it can only respond correctly if it is in precisely one physiological state (139, 140).

**Figure 1:**
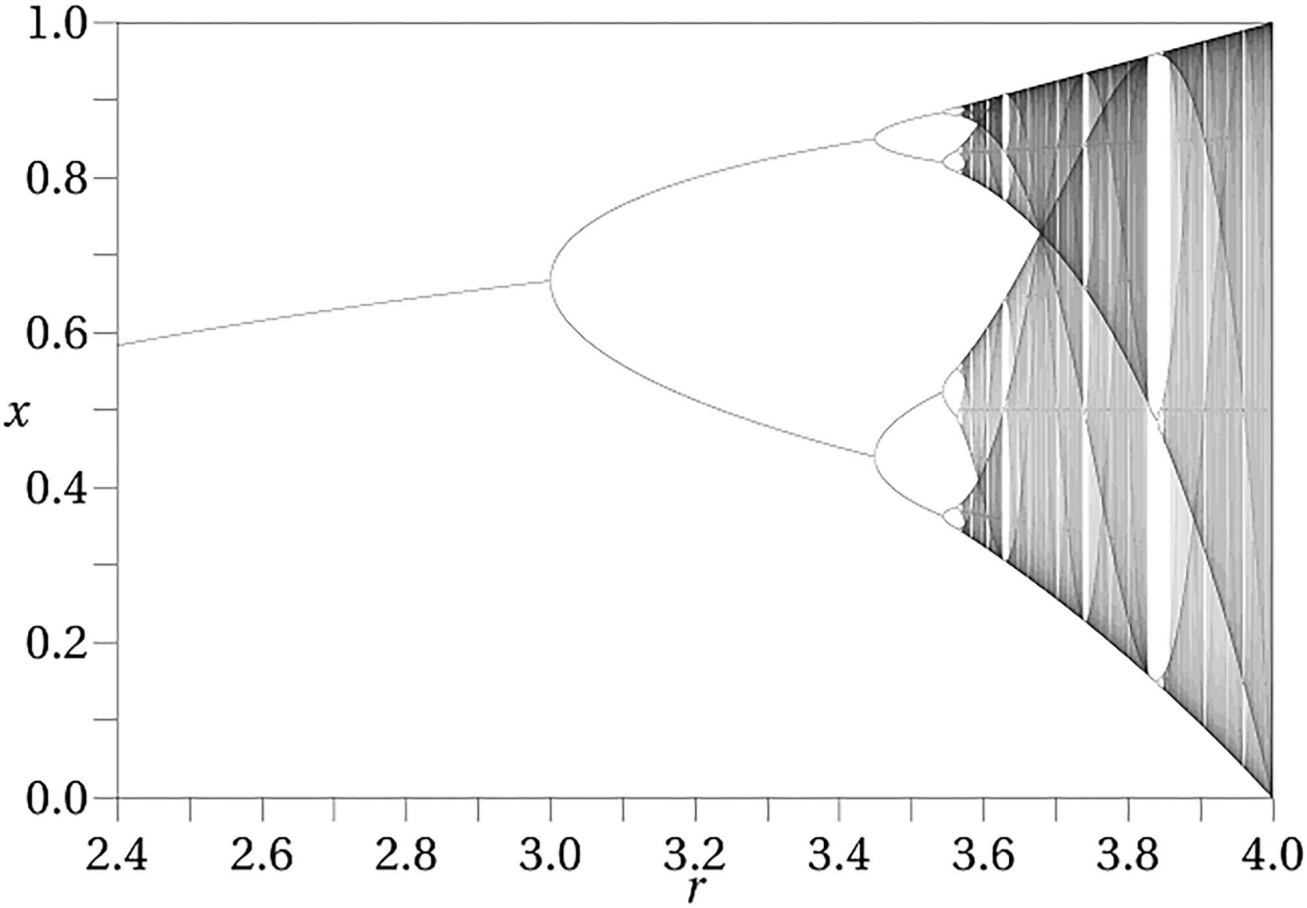
Bifurcation diagram for the logistic map. The diagram exhibits the attracting set as a function of the control parameter μ.

The iterated logistic map is a simple discrete time dynamical system which illustrates the notion of a dynamical phase. The logistic map is defined as*f* (*x*) = μ*x*(1 − *x*) where x lies in the real interval [0, 1] and μ lies in the real interval [0, 4]. The dynamical action is generated by iterating this map, so that a trajectory with initial state x is given as *x, f*(*x*), *f* (*f* (*x*)), *f* (*f* (*f* (*x*))), … The parameter μ is a control parameter, held fixed for a given trajectory. Changing its value results in a new dynamical system. It can be thought of as representing the internal conditions of the system. Figure 1 shows the points that make up the limiting trajectory as a function of μ. As μ increases from 0 to 4 this limiting trajectory changes from a single point, to two points (period 2), to four points (period 4) and so on indefinitely (called a bifurcation sequence). In the initial stages, there is a range of values of μ over which the limiting set has the same structure, although as μ increases these intervals become ever smaller until eventually they become single points and chaos ensues. Each non-zero interval represents a dynamical phase, a region over which the character of the dynamics remains constant. The specific trajectories change, but the qualitative form of the trajectory (here, periodicity) remains constant. After the transition to chaos is crossed there are regions in which there are no longer dynamical phases, although there are still dynamical states, each corresponding to a given value of μ.

Unfortunately there is another meaning of phase which has been used to define dynamical phases in neurodynamics and which must be distinguished so as not to cause confusion. The second definition of phase derives from the study of waves. If one compares two waves of identical frequency it may appear that the time course of one wave seems to be delayed by a certain amount relative to the other. This delay is called a phase difference. Describing the first wave as *A* sin(ω*t*), the second wave can be described as *B* sin(ω*t* + ϕ) where ϕ is the phase (or phase difference). Surface recordings from large neural ensembles display oscillating patterns that resemble waves. Wave-like synchronization of the activity of collections of neurons has long been proposed as fundamental to neural information processing (141, 142). Freeman was one of the first to suggest that phase relationships in EEG signals could be used to define a concept of *dynamical* phase in the brain and that such phases could be fundamental to information processing. Individual dynamical phases could be recognized by the phase cones (143) associated to a collection of neurons. Within a phase, the phase cones are narrow, suggestive of highly synchronized neural activity, while during transitions between phases they expand. Freeman showed such changes in surface EEG recordings of the olfactory system and showed that different phases corresponded to different odorants with transitions between them associated with a divergence in the phase cones.

Freeman developed these ideas into a theory of mind (143–146) and introduced the idea of patterned attractors (143). A more general form of synchronization has been observed between complex systems and their environments, without the need for synchronization among the individual elements of the systems. This has been termed transient induced global response synchronization (TIGoRS) (147–150) and is particularly relevant to open dynamical systems.

The identification of dynamical phases naturally leads to the study of transitions between dynamical phases and their signatures (151, 152). One approach to such transitions is through the study of intermittency and edge states (58–60, 153). Evidence for phase transitions can often be found in the distribution of fluctuations and rare events (138). Fluctuations within dynamical phases should not alter the geometry of the trajectories, and so are expected to appear like simple noise, typically following a Gaussian distribution. At the point of transition between dynamical phases, fluctuations can be expected to take the system into distinct dynamical phases, transiently generating distinct dynamical signatures rather than simple noise. Under such conditions, fluctuations will often follow a power law distribution, suggested by the occurrence of black swan or extreme events and heavy tails.

Critical points or regions are of interest because distinct dynamical phases lie close to one another in the control parameter space, and thus small perturbations can shift the system quickly from one dynamical phase to another. Freeman suggested that brains utilize homeostatic mechanisms which keep them functioning nearby to a critical region, which permits rapid shifts between dynamical phases in response to environmental contingencies (154). This has spawned research to identify signatures of criticality which could be used as markers of transitions (155–159). There have been attempts by clinicians to try to use some of these markers to provide an “early warning sign” for when a patient is at risk of transitioning into a clinical episode. Some candidate early warning signs are critical slowing down in depression (160) and sudden gains and transient spikes in PTSD (161) and OCD (162). Schiepek and Strunk (163) described two measures useful for identifying critical fluctuations and phase transitions in clinical time series data (Fluctuation intensity F, Distribution D, and Dynamic Complexity FD). The idea of criticality has been applied in psychotherapy (162, 164), Alzheimer's disease (165, 166), chronic disease (167). In addition there have been attempts to use biomarkers of transitions to assist in the prediction of transitions (167, 168).

The characteristics of criticality in the nervous system may not follow the standard profile. In Beggs and Timme (169), cortical motor neurons revealed surprisingly weak correlations but wide dispersion, contrary to standard models of criticality (170). This suggested a second form of criticality dominated by inhibition yet nearly unstable due to heterogeneous connectivity (170). The idea of a subcritical state has been proposed to allow for information processing flexibility while avoiding pathologies such as seizures (171).

An animal model of OCD exhibited dynamical phases (98). Single injections of the D2/D3 agonist quinpirole increased locomotor activity while repeated injections over the long term resulted in several persisting changes: rats returned to two sites in the environment approximately five times more often than controls, the recurrence time to these two sites was ~1/20th that of the controls and the dwell time increased, Lempel-Ziv complexity declined [0.95 ± 0.16 (subjects) vs. 1.20 ± 0.01 (controls), *p* < 0.001], and entropy declined [0.709 ± 0.005 (subjects) vs. 0.888 ± 0.003 (controls)]. Chronic quinpirole injections appears to induce a distinct dynamical phase. This appears in keeping with the FET model which attributes a role for dopamine in motor tempo, plasticity and orientation to probabilities.

Mental states have often been modeled as dynamical attractors. The attractor approach, however, explicitly *ignores* the initial transient behavior and only focuses upon asymptotic or very long term behavior (i.e., where it ends up after it *settles down*). The bifurcation diagram shown in Figure 1 depicts the attractors for the logistic map as a function of μ (not trajectories). In the conception of dynamical phases of Freeman and many others (144–146), these attractors play the role of dynamical phases, and transitions occur as a result of perturbations of the states, resulting in the system transitioning to a new trajectory.

The fundamental flaw in the attractor approach as applied to behaving systems, particularly neural systems, is that the dynamic, that is, the function *f* from which the time evolution is generated, is not isolated. Indeed a fundamental characteristic of neural systems is that they are dispositional, meaning that the dynamic itself changes as a result of changes in environmental conditions, both internal and external. The classic example of this is the lobster stomatogastric ganglion, a 30 or so neuron network which undergoes functional rewiring as a result of changes in the hormonal environment as food is processed through the lobster gut (172). This single network is able to carry out the roles of many different networks through this functional rewiring. These dispositional interactions are ubiquitous in nervous systems. The majority of receptors are of the g-protein coupled variety, which alter membrane or receptor responsiveness to depolarizations induced by neurons coupled through ligand channels. Thus, neurons which act upon g-protein coupled receptors alter the dynamical response of their target neuron, thereby altering its disposition to respond to stimuli (19).

The phase transitions which now occur can come in many forms: transitions between attracting states of a fixed dynamics, transitions between attracting states of different dynamics, a combination of both types, or in the worst case, transitions between dynamics without relaxation to an attracting state of any of the individual dynamics, instead being transitions between attractors on a space of dynamics or possibly even higher level emergent structures. The complexity of this problem can be illustrated with a simple example. Let us return to the simple logistic map and consider three situations in which two logistic maps are coupled to one another. In the first case (forcing), the output of one map serves as a perturbation of a state of the second map. This can be described as

System 1: *f* (*x*) = μ*x*(1 − *x*)
System 2: *g*(*y*) = ν*y*(1 − *y*) + ε(*x*)

In the next two scenarios (dispositional), the output of one map alters the control parameter of the second map, thus dispositionally driving the dynamics of the second system. This can be described as

System A: *h*(*w*) = λ*w*(1 − *w*)
System B:*k*(*z*) = *g*(*w*)*z*(1 − *z*)

**Figure 2:**
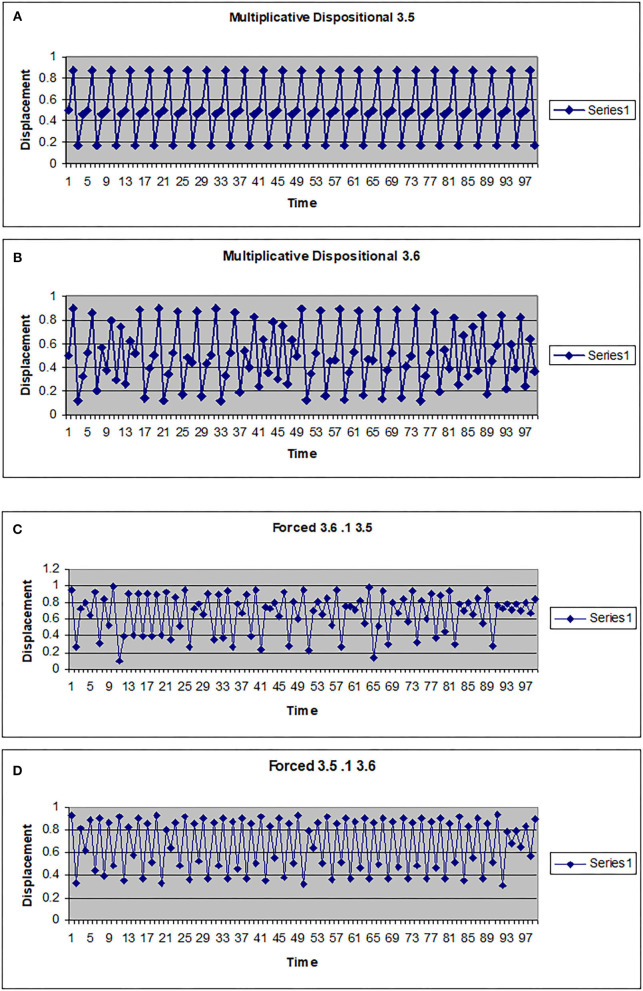
Time series for a logistic map with different couplings. **(A)** Multiplicative dispositional map f(x)=4yx(1-x) where y is the output from g(y)=3.5y(1-y). **(B)** Multiplicative dispositional map f(x)=4yx(1-x) where y is the output from g(y)=3.6. **(C)** Forced map f(x) =3.6x(1-x)+0.1y where y is the output from g(y)=3.5y(1-y). **(D)** Forced map f(x) =3.5x(1-x)+0.1y where y is the output from g(y)=3.6y(1-y).

**Figure 3:**
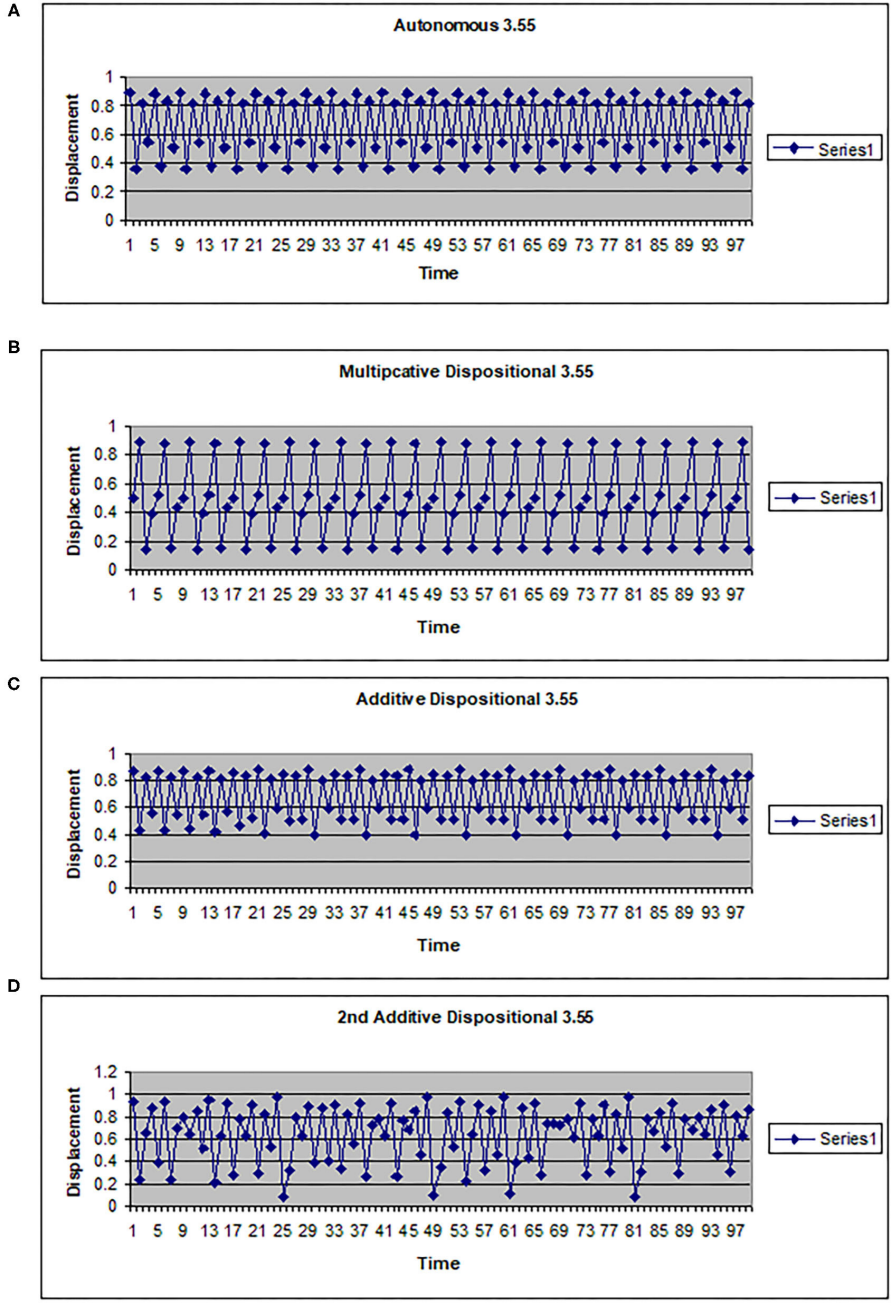
Time series for a logistic map with different couplings. **(A)** Autonomous logistic map f(x)=3.55x(1-x). **(B)** Multiplicative dispositional map f(x)=4yx(1-x) where y is the output from g(y)=3.55y(1-y). **(C)** Additive dispositional map f(x)=(3+y)x(1-x) where y is the output from g(y)=3.55y(1-y). **(D)** 2^nd^ additive dispositional map f(x)=3.5 +0.5y)x(1-x) where y is the output from g(y)=3.55y(1-y).

The map g can be multiplicative (*g*(*w*) = *kw*) or additive (*g*(*w*) = *a* + *hw*). In Figures 2, 3 the driving system is an autonomous logistic map. The values (3.5, 3.55, 3.6) refer to the value of μ used to simulate the driving system. μ = 3.5 corresponds to a period 4 system, μ = 3.55 is also a period 4 system but more widely spread on the interval [0, 1], while μ = 3.6 is approximately a noisy period 14 system. In Figure 2, the upper two graphs illustrate multiplicative dispositional interactions for λ = 3.5, 3.6, respectively and *k* = 4. The bottom two graphs illustrate forcing interactions with ν = 4 and ε = 0.1. The upper shows a μ = 3.6 system being driven by a μ = 3.5 system, while the lower shows a μ = 3.5 system being driven by a μ =3.6 system. Note that when the central system is periodic [multiplicative 3.5 and forced (3.5, 0.1, 3.6)], the resulting time series appears to be periodic or nearly periodic. Likewise when the central system is noisy the resulting time series appears noisy. However, Figure 3 shows that this is not necessarily the case. The top graph shows the time series for an autonomous system with μ = 3.55, a 4 period system. The second and third graphs illustrate multiplicative dispositional interaction (*k* = 4) and additive interaction (*a* = 3, *h* = 1). All three show periodic behavior. However, the bottom graph illustrates an additive interaction with *a* = 3.5 and *h* = 0.5. This time series is clearly not periodic. The reason is that the driving function *g* now takes the control parameter well into the chaotic range for at least part of the time, introducing an intrinsic stochastic element into the dynamics.

The point of these illustrations is to show that knowledge of the time series alone will not yield insights into the nature of the underlying dynamics without an underlying model. In multi-scale, multi-level systems it is necessary to examine the system at all dynamically relevant levels if reasonable and effective models of the dynamics are to be obtained. There needs to be an on-going interaction between theory and experiment, so each can guide the other into a deeper understanding.

Another fundamental issue, which has received scant recognition in the literature, is the presence of contextuality. At its most basic level, this is a recognition that the behavior of a system depends in part upon the behavior of its immediate environment. At a deeper level, it reflects the fact that behavior is generated, not elicited. There is no fixed internalized representation or program which is activated in response to some event in the environment. Instead, every response must be generated “on the fly,” often using different neural systems to do so. At best, internalized information acts like a play; it broadly specifies the outcome but not the participants. The generative and interactive nature of behavior results in a deeper form of contextuality which was recognized in physics nearly a century ago in the probability structure of quantum mechanics (173–175). It was also observed in psychology, particularly in decision theory, but only recently recognized as contextuality. In recent years it has been recognized as contextuality (176–178). In fact contextuality of a form similar to that observed in quantum mechanics has been observed in psychological experiments at both the social (179) and the individual level (180). This is a profound observation, because it demands a re-thinking of the use of probability theory in describing, modeling and analyzing behaving systems. Problems in the naïve application of Kolmogorov probability theory to biomedical phenomena have already been noted (181). The recognition of contextuality demands a shift toward the use of non-Kolmogorov probability theory such as Khrennikov's contextual probability theory (173) or Dzhafarov's contextuality by default model (176). A failure to take contextuality into account means that inferences drawn from the use of statistical analysis risk being erroneous, particularly in any situation in which assessments involving multiple conditions are carried out, something which is common in the biomedical sciences. There are several excellent books on the subject of non-Kolmogorov probability as its applications in psychology, economics and physics (174, 175).

## Dual Determinants of Behavior: Energy and Information

For most of its history, the physical sciences have been dominated by the concept of energy. In ancient times this was represented by the phenomenon of fire. By the time of Leibnitz it had become vis viva, the live force. In the Nineteenth century the modern concept of energy began to emerge along with its various manifestations, transformations and constancy. These ideas became formalized in the laws of thermodynamics, the Hamiltonian function (total energy), the Lagrangian (difference between kinetic and potential energy) and the principle of least action (the time integral of the Lagrangian along a trajectory takes an extremal, usually minimal, value) (182).

In the latter half of the Twentieth century a new construct arose: information. Several disparate areas of research contributed to this—ethology, semiotics, biosemiotics, computation theory, semantics, automata theory, communication theory (5, 183, 184). Shannon's concept of information (183) played a crucial role, and its intimate connection to the notion of entropy in physics brought it to the attention of mathematicians and physicists. The development of the digital computer subsequently led to the information age.

Physical systems are described almost entirely by either their Hamiltonian or Lagrangian and follow the principle of least action. Biopsychosocial systems, however, are only partly determined by energy. Indeed most biopsychosocial systems are open systems, continuously interacting with their environment while maintaining a fairly constant throughput of energy sufficient to carry out any necessary behavior. Instead, the behavior of biopsychosocial systems is also determined by *information*, usually in the form of *patterned* interactions.

Biopsychosocial systems exhibit dynamical features that render simplistic, energy dominant, deterministic models at best cartoon stereotypes. Gerstein and Mandelbrot (185) showed that the release of neurotransmitters was a stochastic process, and Shadlen and Newsome (186) showed that the neural spike train response to a given stimulus was also stochastic. They suggested that a collective of neurons provided, at best, only a noisy estimate of a wave. This suggests that the “waves” that are considered essential to neural information processing do not actually exist at the level of individual neurons but instead represent emergent phenomena at the level of neural populations. Moreover, the individual neurons taking part in the processing of some particular information, such as spatial location (187) or long term memory (188, 189) are fungible, meaning that they may take part at one time when processing information but a different collection of neurons will take part when the same information is processed at a later time. Any dynamic serves more as a play, with individual neurons serving as players. The generative nature of neuronal dynamics has already been mentioned. A crucial issue is how to keep such a system stable.

Energetic systems are stable by virtue of being in a local energy minimum. An information system is in continuous interaction with a dynamic environment, resulting in adaptations over time. There is no analog of energy, no minimum principle. Instead one observes a consistent coupling of environment and system responses. One may think of one pair of system (*S*) and environment (*E*) transients as giving rise to the next pair. Formally this can be written as (*S*_1_ : *E*_1_) → (*S*_2_ : *E*_2_). Such a relation, sometimes called a production rule, bears a similarity to an if-then statement in formal logic.

In mathematical logic, the logical equivalent of a dynamical trajectory is a sequence of formal deductions from a fixed set of axioms. The axioms are analogous to the initial conditions of a dynamical system. The rules of inference are analogous to specifying the dynamics of the system. Logical consistency, requiring that deductions do not lead to contradictions, is akin to dynamical stability, since inconsistency means that anything can be derived. For an information driven system, consistency must be supplemented with additional criteria—the behaviors must generally follow some kind of script, schema, expectation, functionality, or ecological validity. The rules of mathematical logic can be expressed as production rules. This idea can be expanded to consider production rules more generally and require that these rules satisfy additional criteria relevant to the particular information setting.

Dynamical systems can also be described in terms of production rules. For example consider a field *f* which satisfies a differential equation of the form

$$\begin{array}{l} \frac{\partial L}{\partial f}-\overset{n}{\underset{i=1}{\sum }}\frac{\partial }{\partial x_{i}}\frac{\partial L}{\partial \left(\partial f/\partial x_{i}\right)} \end{array}$$

where *L* is the Lagrangian for the system. Such a partial differential equation may be converted into a production rule by first transforming it into an integral equation using the method of Green's functions. For suitable boundary conditions one can construct a propagator *K*(*t*, **x**:*t*_0_, **x**_**0**_) which can be thought of as propagating an effect (energy, information etc.) from one spatio-temporal point to another. The function *f* can be constructed from an integral equation

$$f(t,\text{x})=\int K(t,\text{x}:t^{\prime },{\text{x}}^{\prime })f(t^{\prime },{\text{x}}^{\prime })dt^{\prime }\;d{\text{x}}^{\prime }$$

The propagator labels edges in a causal graph which represents information flow in this system. For example we have edges of the form $\Psi \left(t,\text{x}\right)\overset{K\left(t_{1},{\text{x}}_{1}:t,\text{x}\right)}{\to }\Psi \left(t_{1},{\text{x}}_{1}\right)$. In a continuous system there would be an infinitude of such edges but as one will see, the Process Algebra approach described below replaces the continuous system with a finite, discrete system while effectively generating more or less the same function.

Dynamical systems concepts have generally focussed on autonomous systems, that is, systems that are isolated from their environment (although there is a rich engineering literature on control theory and a subfield within physics dealing with open systems). Autonomous and open systems behave in distinctly different ways. Understanding these differences is vitally important for understanding behavior. To illustrate this in the context of behavior, consider a classical dynamical systems model, the cellular automaton, in which a discrete and finite collection of interacting cells, capable of expressing a finite set of states and following a fixed set of rules can generate a variety of spatiotemporal patterns. These automata have been subdivided into 4 classes according to the symmetries exhibited by their (autonomously) generated patterns: fixed point, periodic, complex and chaotic. In the presence of external perturbations (akin to sensory inputs), entirely novel behavior emerges (147, 148, 190, 191). One striking phenomenon, important for understanding neural information processing, is transient induced global response synchronization (TIGoRS) (148, 191). In this paradigm, a fixed spatio-temporal pattern (whose spatial extension matches that of the automaton and temporal extension is arbitrary) is sampled randomly at a low fixed frequency, usually 5–20% of the pattern cells. These pattern samples are then fed into the cellular automaton at corresponding spatio-temporal points, thereby perturbing the state of each cell (for simplicity, most often changing the state of the input cell to match that of the pattern sample—called recognition mode).

**Figure 4:**
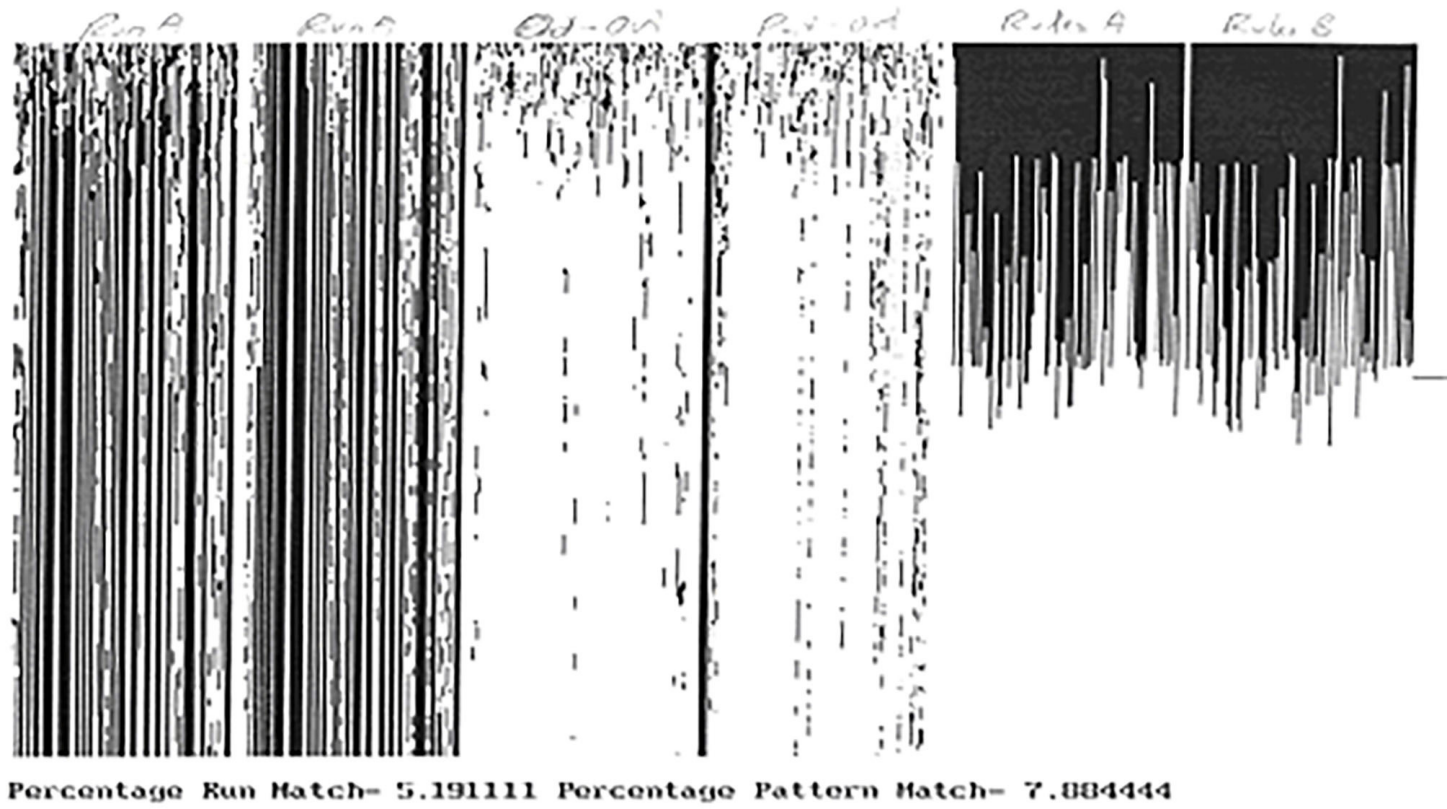
One hundred cells cocktail party automaton simulated with two distinct 10% random samplings of a fixed pattern (100 cells × 450 time steps). Run A and Run B shows the automaton outputs. Out-Out shows discordance between Runs A and B. Out-pat shows discordance between Run A and the pattern. Rule A and B show the distribution of rules across the automaton at end of runs A,B respectively. The individual rules are numbered from 0 to 255.

For example, a cocktail party automaton is an adaptive cellular automaton in which the rules of each cell are allowed to adapt at each time step depending upon the response of a majority of cells having the same neighborhood state. Figure 4 illustrates this for a 100 cell automaton simulated for 450 time steps. From left to right the figure shows the output of the automaton from two different initial conditions and under two different samples at the same frequency (here 10%), followed by the discordance between the two outputs followed by that between the first output and the pattern, and then the rule configurations and then end of the two runs. Note the very low discordance between the output and the pattern. A random 10% sample of the pattern enabled the automaton to reproduce the pattern to nearly 93% accuracy.

Several things can be noticed in this situation. First of all, there is, in general, no apparent relationship between the trajectory of one cell and that of any other cell. In particular, the kind of synchronization of trajectories of individual cells suggested in neural systems seldom occurs. Second, the symmetries of the autonomous system are generally broken, sometimes to the point that the subsequent global trajectory of the automaton appears random. Third, depending upon the manner in which the input sample alters the local state of a cell, the global output of the automaton may appear similar to the pattern being sampled. This may occur across a broad range of initial conditions (191), and even under conditions in which the rules which govern the transitions of the individual cells are allowed to adapt over time (147, 190), and it is important to note that the input sample varies each time. Indeed the presence of fast adaptation appears to facilitate this synchronization between the sampled pattern and the global automaton trajectory.

TIGoRS refers to the appearance of synchronization between the *global* trajectories of a system, across multiple initial states and local rule configurations, irrespective of any synchronization between *individual* cells. TIGoRS results in a stable coupling between sets of environmental transients and sets of system responses. This kind of response is much more robust than that necessary for phase-locked synchronization of trajectories of individual cells and more reflective of the fungibilty, generality, transience and emergence which characterize neural systems.

TIGoRS can be described using production rules. One may think in terms of a stimulus-response system, and describe the relationship as $S\overset{k}{\to }R$ where *S* is the stimulus transient originating in the environment, *k* is the sampling rate and *R* is the response of the system. In the presence of TIGoRS it is possible to replace both *S* and *R* with sets of stimuli and responses (thus replace individual behavioral acts with behaviors) and preserve the production rule structure. More precisely, one should consider pairs (*S,R*) and production rules of the form (*S*_1_, *R*_1_) → (*S*_2_, *R*_2_), which one will recognize as meta-level production rules, that is, production rules over collections of production rules.

The phenomenon of TIGoRS is important for several reasons. First it suggests that, from an information point of view, the important object for study is not the individual state but rather the collection of global spatio-temporal transients, corresponding to the environmental and system behaviors. Second, it shows that information processing and response generation can occur “on the fly” and be generated through interactions; it does not need to be specified in advance as some kind of internalized representation or response script/program. Third, it establishes a meta-level relationship between environmental transients and system transients. This higher level relationship takes the form of a stimulus-response relationship. A consequence of this is that an aspect of information flow is now introduced into the dynamics of the system-environment complex.

Most of the focus in neural and network dynamics has been on the synchronization of activity of individual agents within the system. As described previously, this idea formed the core of Freeman's work on dynamical phases (142–146) and has been extensively studied in the neuroscience literature, becoming a dominant paradigm. Synchronization, however, is not so relevant at the macroscopic level. Workers in ant colonies do not synchronize their behavior, they cooperate. Workers in a factory too do not synchronize, they cooperate. Mass action appears to be important in both neural and ant behavior (142, 192, 193) but amplitude-phase synchronization is not necessary for its implementation, only cooperation with coherent timing. Natural patterns do not exhibit global synchronization of elements, rather they exhibit regions of local homogeneity within global heterogeneity organized in a coherent pattern. Just as periodicity and recurrence are different, but related constructs, so are notions of synchronization and cooperation/coherence. In TIGoRS, information is expressed through global cooperation and coherence, not amplitude-phase synchronization.

The idea of transient in the definition of TIGoRS should not be equated to the concept of transience. In the study of dynamical systems, many phenomena exhibit transience, meaning that they exist for a finite duration and then disappear, possibly to return at a later time, possibly never. Intermittency is characterized by its transience. So is the concept of transient chaos (194–196). In the study of these phenomena, the central object, a state of the dynamical system, represents a configuration of its elements at a single point in time. Transients, on the other hand, are finite, non-zero duration trajectories of dynamical systems. This has the effect of temporally discretizing the original dynamical system and making spatio-temporal *patterns* the central object of study, rather than individual states. The study of synchronization in networks advances (58, 197, 198) even if it should be thought of as a special case.

This intriguing aspect formed the basis for defining a class of dynamical systems called *dynamical automata* (147, 199) and what Lumsden and Dufort called *Sulis machines* (200). These are essentially automata which act on sets of suitably defined spatio-temporal transients. However, the dynamics of the automaton itself is defined upon a set of more primitive states and the spatio-temporal transients are defined upon these states. The dynamical automaton is actually an emergent dynamical system which supervenes upon the lower level state dynamics. Its existence hinges upon the relationship between these spatio-temporal transients, which serve as inputs or perturbations to the underlying automaton and as the subsequent transient dynamical responses of the automaton. At the higher level its behavior can be described via production rules. At the lower level one has the usual cellular automaton rules. The existence of TIGoRS provides one mechanism through which a simple automaton such as a cellular automaton or neural network can become a dynamical automaton at a higher spatio-temporal scale. Thus, one obtains a dual system which has a physical or energetic aspect at the lower level and an informational or semantic aspect at the higher level. This is valuable because it not merely allows for the modeling of multi-scale, multi-level, multi-component systems, but it provides for the unification of two seemingly disparate modes of describing reality, the physical and the psychological. These two domains move us to the study of process.

## Process and the Process Algebra Approach to the Continuum

It should be evident that the dynamics of the continuum between temperament and mental illness, and that underlying biopsychosocial complexity, will not be faithfully addressed using models based upon the traditional dynamics of structurally stable, closed physical systems. Instead there is an urgent need to develop new mathematics, new physics, new experimental paradigms, in order to proceed into the depths of these systems. The previous sections have described many of the characteristics of the dynamics underlying biopsychosocial complexity—transience, emergence, contextuality, interactions, generativity, fungibility, openness, and a fundamental role of information.

Conceptual thinking derives great benefit from the use of metaphors—using similarities between the system of interest and a second, presumably simpler or at least better understood system—to provide guidance in creating conceptual and predictive models and designing experiments. Two metaphors appear promising for understanding the dynamics of the continuum. The first comes from meteorology (43), a field which deals with dynamics across multiple time scales (very short, short, and long duration), across multiple spatial scales [from that of a few meters, to that of a political county and up to that of the entire earth (and sometimes beyond)], and involving systems of varying scale and complexity (from waterspouts to thunderstorms to frontal systems to climate zones). The dynamics of meteorological systems possess most of the characteristics of the continuum except, perhaps, for a role for information. In forming the metaphorical linkage, note that behavior and behavioral acts occur locally and over relatively short time scales. They form the basic phenomenological element of the continuum. They thus bear a similarity to small, local weather systems, which are the fundamental entities studied in meteorology and which are also of relatively small physical and temporal scale, and local. Note that both types of entities are transient, emergent, contextual, interactive, generative, fungible and open. They differ primarily in the role of information, which is fundamental to behavior but not so for meteorological systems (so far as we know). Nevertheless, meteorological systems display many of the same characteristics at least in so far as the flow of energy through these systems is concerned. Indeed, intermittency has been studied in climate models in shifts between the snowball climate and the warm climate (153). Moreover, the metaphor goes both ways, with climate modelers describing transitional states as “Melancholia” states (in homage to the eponymous film which is itself a meditation on despair) (201).

In this metaphor, therefore, one can associate behavior and behavioral acts to local weather. Continuing the metaphor, temperament, which is normative and refers to long duration patterns of behavior extending across a range of different contexts, can be associated to climate, which describes long duration patterns of local weather across a range of seasonal contexts. Acute mental illness, which consists of short to medium duration patterns of maladaptive, dysfunctional, pathological patterns of behavior can be associated to frontal systems and storms, which are short to medium duration patterns of dysfunctional, pathological weather. Chronic mental illness, which consists of long duration patterns of altered or dysfunctional, pathological behavior can be associated to climate change (13, 14). The purpose for exploiting this metaphor is not to draw on the specifics of the formal models, the differential equations and such used to describe these meteorological phenomena, which are clearly inappropriate, but rather to draw on the qualitative dynamical constructs and in particular various time series methodologies for identifying various dynamical phases and their transitions, and in particular, identifying markers of critical states.

There is a deeper layer of complexity in this metaphor which can also be exploited. The study of weather and of climate is not merely a study of atmospheric parameters such as temperature, pressure, humidity, wind sheer and so on. It also depends upon non-atmospheric parameters such as terrain, distribution of water and vegetation, albedo, ozone and greenhouse gas distribution and composition, solar cycle and so on. In the parlance of dynamical systems theory, the atmospheric variables are the state variables, the observables. The non-atmospheric variables collectively form the boundary conditions, or the control parameters. In dynamical systems theory the dynamic is typically described by either a differential or an integral equation and trajectories are functions of time which satisfy these equations. However, in general, such equations cannot be solved without first specifying the initial and the boundary conditions. The initial condition refers to a specification of the form of the t=0. The boundary conditions generally refer to specifying the form of the solution as *t*, **x** → ∞ and sometimes on other spatiotemporal regions as well. However, there are often additional parameters which form part of the equation, explicitly or implicitly, which can vary depending upon local circumstances, and which determine the ultimate landscape of trajectories. These boundary conditions may themselves change over time but usually at a time scale which is much longer than that of the phenomena of interest. One boundary condition, one that is subtle and frequently ignored, is the choice of the geometry of the space of dependent variables. This is the mathematical analog of the terrain in meteorology. This space is generally made explicit in the integral equation specification since it defines the region of integration, but it frequently is left implicit in the differential equation form. Usually this space is taken to be a 3-dimensional Euclidean space, which in the meteorological setting means a flat Earth. While that may appeal to modelers and certain members of the public, it makes a rather poor fit to reality.

Either the differential or integral equation formulation can be represented mathematically as a functional, that is, a function of functions, in the form. This can be expanded by making explicit the internal parameters and the boundary conditions and write this as

$$\begin{array}{l} F\left(f|a_{1},\ldots ,a_{n};g_{1},\ldots ,g_{k}|\alpha_{1},\ldots ,\alpha_{j};\text{X}\right)=0 \end{array}$$

where the *a*_*i*_ are tunable constants, *g*_*i*_ are forcing functions (such as potentials and external forces), α_*i*_ are external distributions (such as water, vegetation) and **X** is the space of the dependent variables (terrain). For example the Schrödinger equation can be written as,

$$\begin{array}{l} i\hbar \partial \Psi /\partial t+\frac{\hbar^{2}}{2m}\nabla^{2}\Psi +V\Psi =0 \end{array}$$

or as *f* (Ψ|*m*; *V*|**X**) = 0 where *m* is a tunable constant (mass), *V* is a forcing function and the space **X** is implicit.

Temperament and mental illness have been suggested to represent different degrees of dysbalance among the NBRS. One should not misconstrue this to imply that there are simple dimensional scales along which one can define temperament for low values and mental illness for high values. The use of the term dysbalance is meant to suggest changes in the dynamics of the interactions among the various NBRS. In other words, temperament and mental illness are thought to represent different regions in the landscape of all possible dynamics linking the NBRS to one another. They are to be viewed as distinct dynamical phases. Logically, therefore, there should exist distinct types of dynamical phase transition linking them. There is an additional subtlety, which is that temperament does not cease in the presence of mental illness. One does not supplant the other, as a simplistic dimensional model might suggest. Instead temperament persists, but as the results of the FET studies (12, 21–24) illustrate, distinct patterns in temperament profiles seem to appear in the presence of mental illness. This suggests that although temperament and mental illness may supervene upon the same set of NBRS, they are manifesting subtly different dynamical characteristics.

In the meteorological metaphor, temperament and acute mental illness are considered analogous to climate. However, climate has many determinants which make up the boundary conditions for the meteorological equations—terrain, surface constitution, vegetation cover, albedo and so on. Some of these are more enduring than others. Some are more readily altered than others. Since temperament exists from birth, prior to socialization and in the absence of illness, particularly intracerebral trauma and endocrinological disorders, it persists relatively unchanged throughout the life span. This suggests that temperament may have more to do with the enduring boundary conditions such as terrain, surface constituents, perhaps vegetation cover and the like. Chronic mental illness may affect those, but since in many cases it may be ameliorated or even reversed, this suggests that it may be related more to the more changeable boundary conditions such as vegetation cover, albedo, ozone and greenhouse gases concentration and so on. The questions now becomes whether it is possible to observe in the structure and dynamics of the NBRS, characteristics which play the role of dynamical boundary conditions, and whether it is meaningful to separate them out into enduring and ephemeral categories. These are important considerations because it is highly likely that such characteristics will determine distinct sets of biomarkers. The search for appropriate biomarkers which may be used to develop a taxonomy of temperament and mental illness is of pressing concern (202).

Of course what makes this so challenging is that these boundary conditions do not reside in the structure of the space of dependent variables, such as the space-time structure, or the solution space, but rather temperament and mental illness are expressions of higher level dynamics, so that their underlying spaces are landscapes of dynamical systems, each of which possesses its own set of boundary conditions. The boundaries for these higher order dynamical systems are thus space of lower level dynamical systems which possess particular kinds of structure. The study of such complexity has barely begun, but it clearly presents a wealth of opportunity for the development of novel forms of mathematics, physics, biology, psychology, psychiatry, neuroscience and so on. A multidisciplinary collaboration is essential.

The meteorological metaphor captures only the energetic aspects of the continuum. For the informational aspects there is a second metaphor worthy of consideration. This is collective intelligence, the archetypal example of which is the social insect colony (192, 193, 203). At first glance one might think that social insect colonies and NBRS have little in common. But for the point of view of the dynamics of information processing there are striking similarities which may be exploited in developing theoretical frameworks and models.

The ability of a social insect colony to engage in ecologically meaningful behaviors beneficial to the well-being of the colony depends critically upon the effectiveness of interactions between the members of the colony in communicating information about the environment, followed by actions upon that environment which serve to preserve or amplify that information. Communication between ants takes several forms. There can be direct contact between individual ants, such as rubbing antennae on each other's bodies, or the carrying of one ant by another. The bodies of workers may be used to form temporary structures, such as nests in army any (*Eciton burchelli*) colonies or bridges in left cutter (*Atta sexdens*) colonies, which in turn enable other works to carry out essential functions. They may emit local pheromones which are used to signal colony membership and perhaps other roles. Then there are global pheromones which are released into the environment which are used to shape foraging and defensive active. A more subtle form of communication is those stigmergic products, patterned local structures which are used to stimulate specific activities in the construction of nests for example. Honey bees are famous for the waggle dance, used to communicate information about the location of food sources.

One of the main NCRS underlying both temperament and mental illness is the monoamine system. In spite of its relatively small size (having around 250, 000 neurons in the raphe nuclei), the serotonin system plays a fundamental role in structural maintenance and neuroplasticity (204). When the serotonin system is active, it results in a tonic production of serotonin, which binds to 5-HT1A receptors on glial cells resulting in the production of a glial neurite extension factor called S100B. This factor stabilizes the microtubular structure which forms for the cytoskeleton of neurons and astrocytes. During sleep, when the serotonin system is inactive, production of this factor ceases. The result in a reorganization of microtubular structures leading to dendritic reorganization. Every single night, dendrites retract, and the following day extend, giving rise to local neuroplasticity. Azmitia has suggested that the serotonin system plays a holistic role, regulating the function of the nervous system as a whole, even if doing so through local activity (204). It helps to maintain the integration and dynamic stability of the brain as a whole. It is also a plastic system in its own right, adapting to various sensory inputs and glial cell activity, so as to maintain a dynamic homeostasis in response to an ever changing environment. The important point in so far as information processing is concerned, is the dynamic nature of dendritic connections. These are thought to be the site at which information processing takes place within the central nervous system. They form the fine structure of the wiring of the brain. Yet, if this local wiring potentially changes each night, how is it that behavior remains stable at the macroscopic level? Social insect colonies are able to repeat ecologically salient behavior at the macroscopic level even though each time they do so it involves different workers behaving in different ways. Nevertheless, the end result serves the same function for the colony as a whole.

The standard teaching regarding information transmission within the nervous system is that it takes place at the synapse. Ligand gated synapses, which result in direct alteration of the neuronal membrane potential, and G-coupled protein receptors, which alter membrane responsiveness, have been discussed previously (19). Information transmission may occur as a result of mass action, when large number of neurons initiate action potentials in a more or less synchronized manner and impinge on a receiving neuron. This is analogous to quorum threshold decision making in social insect colonies, in which collective behavior on the part of the colony is initiated whenever eliciting behavior on the part of individual workers exceeds a critical threshold.

The volatility of these two forms of synaptic transmission make them poor candidates for the source of the dynamics underlying temperament at least, and possibly for many forms of chronic mental illness as well. In recent years, however, it has been realized that there are many non-synaptic modes of information transmission in the nervous system as well. Volume transmission, the release of neurotransmitters or neuropeptides into the extracellular space which can then diffuse to distant neurons, has been shown to play a prominent role in neurodynamics (205–209), particularly in long duration phenomena such as mood (205). This may be the central means by which serotonin acts (204). Another mode of transmission which has received insufficient attention in psychiatry is via gap junctions, where two neurons (or cells generally) are directly coupled (206). A third mode of transmission which has not been recognized much at all is ephaptic transmission, which occurs between cells which are physically adjacent but not synaptically coupled (209). All of these processes are independent of the dynamics associated with dendrites. As such they may be much less sensitive to fast adaptive changes and learning, and thus present themselves as more likely candidates for the dynamical mechanisms underlying temperament (and perhaps some chronic mental illnesses). These additional forms of information transfer all have counterparts within social insect colonies. Volume transmission is directly analogous to global pheromone secretion. Gap junctions have an analogy in direct touching of individual workers with one another. Ephaptic transmission is analogous to information transfer when large numbers of workers have joined their bodies together to form structures like nests and bridges. Stigmergic artifacts play a role akin to tools, construction artifacts and sociocultural memes in that they involve a feedback interaction between the environment and behavior. Thus, when exploring specifically information flow in NBRS there is much in common with that within collective intelligence systems. The fundamental difference between a collective intelligence such as a social insect colony and NBRS is that the individual agents with the collective intelligence are freely mobile. There is mobility within neuronal assemblies but it takes place over vastly smaller spatial scales and over relatively longer time scales, and mostly at the dendritic level, although a case might be made for mobility of a similar kind occurring at the receptor level, and perhaps the axonal level, albeit there only over quite long time scales. This kind of mobility may play a role in neuroplasticity (and therefore potentially in mental illness) but less so in short term information processing.

The most important implication from all of this is that models of behavior that are based upon analogies to computational theory and thus to digital computers, or to formal logic, or to networks, neural or otherwise, just as models based upon structurally stable, closed dynamical systems, will all fail to capture the deeper levels of dynamics. It bears repeating that the dynamics of NBRS (and of behavior more generally) is transient, contextual, emergence, generative, fungible, open (at a minimum).

Thus, there are two very different metaphors which can provide some inspiration in research into the continuum between temperament and mental illness. They are the meteorological metaphor, which addresses energetic issues, and the collective intelligence metaphor, which addresses informational issues. Linking both metaphors is the concept of process. A.N. Whitehead (210) pioneered the modern concept of process, which he called a “philosophy of organism.” Whitehead considered a process to be a sequence of events having a coherent temporal structure in which relations between the events are more fundamental than the events themselves. In Whitehead's view, process comes first, and material objects are emergent from process. Contrary to the physical world view, becoming, and thus transience, is considered as fundamental to process. Events have a transient existence, coming into being, manifesting briefly, then fading away. The fundamental entities in Whitehead's theory are *actual occasions*, which exist just long enough to *prehend* the previous occasions and form a response, following which they pass out of existence and become data for subsequent events. Information plays a fundamental role and in particular, meaning is necessary to create coherence among events (147–149, 199, 211). Behavior has long been known to be generated “on the fly” by an organism (212). Behavior can thus be considered to be generated by process. On the other hand, behavior itself also generates, and thus behavior itself can be viewed as being a process, which in turn is generated by processes. This is another example of the subtlety associated with the concept of behavior and why a multi-scale, multi-level, multi-system approach is necessary.

Processes *per se* exist outside of space-time, serving instead as generators of space-time and the events within (199, 211, 213). Like the actual occasions that they generate, processes do not move. They instead shift between periods of activity and inactivity, and interact with one another. While active, processes express a propensity to determine differences, manifesting in distinct attributes and functionality, thus according to at least one definition, they are real (211). Processes interact with one another according to their attributes and functionalities and the actual occasions that they manifest, and these interactions are triggered by the manifesting of particular actual occasions.

Trofimova (214–216) has proposed several process algebra based formalisms for describing the principles of transience which govern processes in functional constructivism. Her approach to process algebra uses several functional differentiation classes, a concept of “performance” and several universal process-trends. It applies particularly to complex, adaptive, multiscale systems. In (214, 215) and in particular, (216), Trofimova describes in detail the various functional processes involved in the construction of actions: maintenance, context processing, orientation/expansion of possibilities, emotional dispositions, integration of programming and of experience, storage of “habits.” She describes the dynamic interplay that exists among these different functional “blocks” and then explores the linkages between these different functional blocks and the underlying NCRS that determine them at the neurophysiological level. In particular she demonstrates the ensemble relationships that were proposed in her functional ensemble of temperament model. Functional constructivism provides a promising approach to the identification of relevant biomarkers suitable for understanding and describing the continuum between temperament and mental illness.

A second, complimentary approach is based upon the Process Algebra. It seeks out universal formal characteristics of high level dynamical systems, dynamical phases and dynamical phase transitions. It aims to examine generic mechanisms of energy and information flow in complex adaptive systems. It is not a particular model but instead provides a specific general formal language for describing the actions of processes and their interactions. For the interested reader, mathematical details have been provided in the Appendix. The Process Algebra approach has been applied to problems in the foundations of quantum mechanics and is now being applied to neuronal and collective intelligence systems. The Process Algebra was inspired by combinatorial games (217–219), which also provide an example of the generation of a system. In the Process Algebra language, processes generate discrete and finite sets (causal tapestries) of primitive elements called *informons* (short for informational monads, or actual occasions) from which continuous systems arise through emergence. A process P generates a set of informons *I* = {*n*} (called a causal tapestry). Each informon *n* is associated with a causal manifold interpretation **m**_*n*_ (which can represent a space-time location or a stave value), a local Hilbert space interpretation ϕ_*n*_(**m**_*n*_:**z**) (which serves as an interpolation function) and a local coupling effectiveness Γ_*n*_ (which can represent a state value or a probability value). A global state function for the system is then constructed via interpolation as:

$$\begin{array}{l} \Psi \left(\text{z}\right)=\underset{n}{\sum }\Gamma_{n}\phi_{n}\left({\text{m}}_{n}:\text{z}\right) \end{array}$$

The evolution of the behaving system consists of a succession of generation cycles, each governed by a process. To each process $P_{i}$ there is a global state function Ψ_*i*_(**z**). An evolution of the behaving system $P_{1},P_{2},P_{3}\ldots$ gives rise to a global history function Ψ_1_(**z**), Ψ_1_(**z**) + Ψ_2_(**z**), Ψ_1_(**z**) + Ψ_2_(**z**) + Ψ_3_(**z**), … The behaving system can be described by a collection of processes $\left\{B_{i}\right\}$ and its environment can also be described by its own collection of processes $\left\{E_{j}\right\}$. These couple together, which can be described by some formula in the Process Algebra $F_{ij}\left(B_{i},E_{j}\right)$.

Now the processes for the behaving system and its environment can be decomposed into component subprocess. At a minimum they be expanded into a coupling of three distinct subprocesses:

$A_{k}$, corresponding to afferent processes
$T_{l}$, corresponding to information transport processes
$M_{m}$, corresponding to efferent processes
Thus, each main process can be decomposed as $\left(A_{1},A_{2},\ldots ,A_{k}\right)⨶\left(T_{1},T_{2},\ldots ,T_{l}\right)⨶\left(M_{1},M_{2},\ldots ,M_{m}\right)$.
The behaving system—environment coupling can then be written rather loosely as:

(1) $$\begin{array}{ccc} \left(A_{i}^{ℬ}\right) & ⨶(T_{l}^{ℬ})⨶ & \left(M_{m}^{ℬ}\right)\\ ) & \; & )\\ \left(M_{i}^{ℰ}\right) & ⨶(T_{l}^{ℰ})⨶ & \left(A_{m}^{ℰ}\right) \end{array}$$

Note that each subprocess in the above block can be replaced by an interactive block of subprocesses, corresponding to reducing the scale. Likewise, each block can be replaced by a single process identifier, as can contiguous concatenations of processes, corresponding to increasing the scale. The inherent fractal structure of these spatio-temporally nested processes should be evident. Since each concatenation corresponds to the implementation of a production rule, the flow of information starts to become more evident. Moreover, each process can be interpreted in terms of the informons and causal tapestry that it generates and its associated global history function and thus its links to energetic considerations (linked to its propagator) can be made evident.

Dynamical phases and phase transitions can now be considered according to a variety of different perspectives. They can be sought in terms of geometric attractors at different levels. They can be sought in energetic terms as local minima of a global potential, assuming that the configuration of interactions is conducive to such a generalization. They can be sought in information theoretic/semantic terms in coherent sets of production rules constraining local interactions. Moreover, markers for phases and transitions can be sought as a function of spatio-temporal scale.

Notions of phase at one level may become irrelevant as one moves to higher or lower levels. At the every least the Process Algebra formalism allows for bookkeeping when tracking interactions and levels. However, it is more than that since each process can be replaced by the global Hilbert space interpretation that it generates, which can then be used to carry out specific calculations depending upon the system of interest.

In seeking out biomarkers and statistics, the Process Algebra formalism makes explicit the coupling between the behaving system and its environment and the inherent contextuality involved at every level. This is an essential step in order to move beyond the limitations of current methods.

## Conclusions

Complex systems methodologies offer much to the study of biopsychosocial complexity, whether at the research or the clinical level. Early forays into the use of complex systems methods at the clinical level have already begun to reap some rewards in terms of mood prediction and the prediction of change in psychotherapy. The realization that the dynamics of the biopsychosocial hierarchy is radically different from that suggested by traditional mathematics and physics has yet to reach the majority of clinicians and researchers. Concepts of transience, generativity, fungibility, metastability, emergence, contextuality, non-Kolmogorov probability, information, semiotics are not yet part of the basic teaching into biopsychosocial complexity, yet they are fundamental to its structure. To move forward it is essential that theories and methodologies which explicitly recognize and embrace these concepts be developed and applied both at the research and at the clinical level. The dual structure of biopsychosocial complexity as both energetic and informational has yet to be fully exploited. The continuum between temperament and mental illness is suggested as an area for focus as it embraces the whole of the biopsychosocial continuum, yet is still circumscribed. The Process Algebra is presented here as both a language for describing biopsychosocial complexity and a tool for modeling representative systems. The lack of a direct application of these ideas to psychology is a limitation of this review, but any such application would be purely speculative at this time. It is hoped that this review will underscore the limitations of current methodologies and foster further research. The study of the dynamics of dynamics and of the parallels between neuronal networks and collective intelligence using the Process Algebra is an active focus of research in the Collective Intelligence Laboratory.

## Author Contributions

The author confirms being the sole contributor of this work and has approved it for publication.

## Conflict of Interest

The author declares that the research was conducted in the absence of any commercial or financial relationships that could be construed as a potential conflict of interest.
