# Supplementary material for: The Continuum Between Temperament and Mental Illness as Dynamical Phases and Transitions
Source: Front Psychiatry. 2021 Jan 18;11:614982. doi: 10.3389/fpsyt.2020.614982 (PMC7848037; doi:10.3389/fpsyt.2020.614982)
Supplement: Supplementary file 1 [file Presentation_1.pdf]

Appendix: A Brief Tour of the Process Algebra

This appendix gathers together some mathematical details for understanding the structure of the Process Algebra and of the Process Algebra model of process. It is not for the faint of heart and is intended for the readers who would like to move beyond the conceptual level and use the framework for constructing their own models.

In the Process Algebra model, each process is considered to be a generator of a finite, discrete set of informons. Complex systems often possess state descriptions in the form of wave functions or probability distributions, both of which mathematically form a structure called a Hilbert space. Many types of Hilbert space (and in particular that used in quantum mechanics) have an additional feature which makes them reproducing kernel Hilbert spaces (1). Given a reproducing kernel Hilbert space *H*(*X*) with base space *X*, one can find a discrete subspace *Y* of *X* (sampling subspace), and a Hilbert space *H*(*Y*) on *Y*, such that each function in *H*(*Y*) can be lifted to a function in *H*(*X*) via interpolation. Interpolation means that if $\Psi(z)$ is a function in *H*(*X*), then for each $y \in Y$ there exists an interpolation function $\Psi_y(z)$ on H(*X*) such that $\Psi(z) = \sum_{y \in Y} \Psi(y)\Psi_y(z)$. In general there are usually an infinite number of these sampling subspaces. These interpolations functions are not unique but in the case that the subspace *Y* has the form of a regular lattice the interpolation functions may be taken to be sinc functions ($\sin x / x$) (1). If the subspace has an irregular structure with density matching the Beurling density (2), Fechtinger-Gröchenik interpolation theory may be used instead (1).

In the Process Algebra, the discrete subsets *Y* are considered to be fundamental, their elements representing the actual occasions of Whitehead's process theory. The elements of *H(Y)* are considered to be ontological, while the elements of *H(X)* are derived (emergent) through an (arbitrary) interpolation procedure. The elements of *Y* are considered to be generated by process, **P,** and the value $\Psi(y)$ assigned to a point *y* in *Y* is also generated by **P** by causally propagating specific information from prior actual occasions to nascent actual occasions by means of a causal propagator, **K**. The resulting wave function $\Psi(z) = \sum_{y \in Y} \Psi(y)\Psi_y(z)$ is thus emergent.

Interactions between processes are conjectured as being triggered by the generation of informons according to the compatibility between the processes. Compatibility between interacting complex systems is an idea first proposed by Trofimova (3). In the current context it can be thought of as a generalization of the idea of coupling factors. Compatibility Ξ(**P,M**) is conjectured to be a function of fixed factors such as mass, charge, coupling constants and of the local compatibilities. The probability of an interaction taking place **Π**(**P,M**) is in turn a function of the compatibility, **Π**(**P,M**) = *χ*(Ξ(**P,M**). The precise form of these functions depends upon the particular case. The Born rule (from which probabilities are derived in quantum mechanics) is expected to arise from these interactions and from the compatibility, but a precise derivation is not yet in hand. If one naively applies the Born rule, then probability will be proportional to the

local process strength. If so then it will be non-Kolmogorov by virtue of the presence of interaction terms

$$\Gamma_n^*\Gamma_n = \sum_{m\in I} K(n,m)^*\Gamma_m^* \sum_{m'\in I} K(n,m')\Gamma_{m'} = \sum_{m\in I} K(n,m)^* K(n,m)\Gamma_m^*\Gamma_m + \sum_{m\in I}\sum_{m'\in I, m\neq m'} K(n,m)^* K(n,m')\Gamma_m^*\Gamma_{m'}$$

Each informon is represented as $[n]<\mathbf{p}_n;\mathbf{m}_n:\phi_n(\mathbf{z});\Gamma_n>\{\mathbb{G}_n\}$ where

1) n is a heuristic mathematical label,

2) $\mathbf{p}_n$ is a structured set of intrinsic properties,

3) $\mathbf{m}_n:\phi_n(\mathbf{z})$ is a pair of extrinsic properties,

4) Γ_n is the local coupling effectiveness,

5) $\mathbb{G}_n$ is a causally ordered collection of informons, with causal metric ρ, called the content. The union of content sets over all informons in the causal tapestry must itself form a causal set (4).

The local process strength at an informon n is given as $\Gamma_n^*\Gamma_n$. The information residing in the informons of the content is utilized by the generating process to create the informon. The intrinsic properties $\mathbf{p}_n$ are attributed to the generating process **P** and imparted to each informon generated by **P**. The extrinsic properties are unique to each informon but are frame dependent. Each informon n is interpreted as a point $\mathbf{m}_n$ (*causal manifold interpretation or embedding*) in some causal manifold ***M*** (which can be thought of in various ways as space-time or as a state space). Its content set $\mathbb{G}_n$ causally embeds into ***M***. Each causal tapestry forms a causal antichain in ***M***, and thus represents a discrete sampling of a spacelike hypersurface in ***M***. Each informon n is associated with a *local Hilbert space interpretation* of the form $\phi_n(\mathbf{r}) = \Gamma_n f_n(\mathbf{r},\mathbf{m}_n)$ (which can be thought of as a wave, or a field, or a local probability), the Hilbert space H(***M***) being that over the causal manifold ***M***. Each causal tapestry I is associated with two different maps: a *tapestry realization* (or allowing a slight misnomer, a *tapestry "wave function"*) of the form $\Omega(n) = \Gamma_n$, and a *global Hilbert space interpretation* over the causal manifold of the form $\Psi(\mathbf{r}) = \sum_{n\in I}\Gamma_n f_n(\mathbf{r},\mathbf{m}_n)$. When the informons of a causal tapestry embed into the causal manifold as a discrete lattice, it is possible to replace each $f_n(\mathbf{r},\mathbf{m}_n)$ by a spatial translation ($T_{\mathbf{m}_n} f(\mathbf{r}) = f(\mathbf{r}-\mathbf{m}_n)$) of a single generic sinc function $g(\sigma,\mathbf{z}) = \sin\sigma\mathbf{z}/\sigma\mathbf{z}$, so that $\Psi(\mathbf{r}) = \sum_{n\in I}\Gamma_n T_{\mathbf{m}_n} g(\sigma,\mathbf{r})$. The lattice spacing must be consistent with the Beurling density (2). Maymon and Oppenheim (5) have shown that non-uniform embeddings still provide a highly accurate approximation using sinc interpolation so long as the spatiotemporal density is large enough. A more realistic model requires the use of non-uniform embeddings and more sophisticated interpolation techniques, such as Fechtinger-Gröchenik theory (1).

Processes possess three additional intrinsic characteristics:

1) r, the number of prior informons whose information is incorporated into an informon *n*. It is also the cardinality of $\mathbb{G}_n$, and the number of short rounds needed to form *n*.

2) N, the number of informons in each generation, and thus the number of rounds and the cardinality of the causal tapestry I.

3) R, the number of informons generated per round. A *primitive* process has R=1. Otherwise the process is *compound*.

The action of a process involves:
1) The assignment of a new informon label
2) The assignment of property set $\mathbf{p}_n$
3) The assignment of causal relations and distances to prior informons
4) The assignment of a content set $\mathbb{G}_n$
5) The propagation of information from prior informons.
6) Determination of local coupling effectiveness by propagating the local coupling effectiveness from each informon in $\mathbb{G}_n$ forward to *n* according to the rule $\Gamma_n = \sum_{m\in I} K(n,m)\Gamma_m$ where the propagator *K* will depend upon the causal distance $\rho(n,m)$.
The propagator will be determined by particle and interacting potentials.

An important concept is that of *epistemological equivalence*. Epistemological equivalence of two processes **P** and **Q** means that their global Hilbert space interpretations, $\Psi^P(\mathbf{r}), \Psi^Q(\mathbf{r})$, respectively, are equal as functions over the causal manifold. In other words,
$$\Psi^P(\mathbf{r}) = \sum_n \Gamma_n f_n(\mathbf{r},\mathbf{m}_n) = \sum_m \Gamma_m f_m(\mathbf{r},\mathbf{m}_m) = \Psi^Q(\mathbf{r}).$$
If two processes are epistemologically equivalent then the specifics of informon generation do not matter in so far as NRQM is concerned. They generate the same emergent wave functions and therefore will yield the same NRQM predictions. This is useful because processes can be modeled heuristically based upon mathematical convenience just so long as they are epistemologically equivalent to any real processes. In particular one can use processes based upon combinatorial games which have particularly valuable characteristics (6-8). Epistemological equivalence may also possess ontological implications in that it might be impossible on principle for macroscopic observers to be able to access information about this most fundamental level. To use a computer analogy, it is generally inadvisable for a computer program to be able to access and change its own code. Perhaps that is the case for nature as well.

The Process Algebra is a formal language and mathematical structure for describing interactions between processes. Epistemological equivalence allows for the use of many different forms of representation for modeling the actions of processes. A particularly fruitful and accessible representation is based on the concept of combinatorial games.

These are generally two player games which may be competitive or co-operative, partisan or non-partizan, with discrete moves, finite resources, a well defined end point, and perfect information. Combinatorial games have been harnessed for a variety of purposes mathematically ranging from recreational play (6) to the generation of mathematical structures in formal logic (7,8), where there have proven to be a powerful tool. Here, the Process Algebra is used to model the flow of information throughout the generation of informons by processes as well as their different modes of interaction and the effects that such interactions have on information flow.

Processes may influence one another in two different ways. The first (*coupling*) involves the generation of individual informons, their relative timing as well as the sources of information which enters into their generation. Coupling results in epistemologically equivalent processes, so properties are unaltered. The second (*interaction*) involves the activation or inactivation of individual processes and the creation of new processes. Epistemological equivalence is broken and properties are altered.

Two processes $\mathbf{P_1,P_2}$ may be independent, meaning that the neither constrains the actions of the other in any way. This relationship is denoted simply by the comma ",". Compound processes (R>1) can be formed from primitive processes (R=1) by various coupling operations. A coupling affects timing and information flow. Two processes may generate informons concurrently (*products*) during each round, or sequentially (*sums*), with only one process generating informons during a given round. Information from either or both processes may enter into the generation of a given informon (free) or information incorporated into an informon by a process may only come from informons previously generated by that process (*exclusive*). This leads to four possible operators:

1: Free sequential (free sum): $\mathbf{P}_1 \hat{\oplus} \mathbf{P}_2$

2. Exclusive sequential (exclusive sum): $\mathbf{P}_1 \oplus \mathbf{P}_2$

3. Free concurrent (free product): $\mathbf{P}_1 \hat{\otimes} \mathbf{P}_2$

4. Exclusive concurrent (exclusive product): $\mathbf{P}_1 \otimes \mathbf{P}_2$

The operation of concatenation is used to denote processes that act in successive generation cycles. Thus $\mathbf{P}_1 \cdot \mathbf{P}_2$ (or simply $\mathbf{P}_1\mathbf{P}_2$) indicates that $\mathbf{P}_1$ acts during the first generation cycle, while $\mathbf{P}_2$ acts during the second generation cycle.

Interactions between processes may activate an inactive process or inactivate an active process. In addition, an interaction among processes $\mathbf{P_1,P_2,..,P_n}$ may generate a new process, $\mathbf{P}$, which can be described in functional form as $F(\mathbf{P_1,P_2,..,P_n}) = \mathbf{P}$. If $\Theta(\mathbf{P_1,P_2,..,P_n})$ describes a coupling among $\mathbf{P_1,P_2,..,P_n}$ then the functional relation may be described using the operation of concatenation, as $\Theta(\mathbf{P_1,P_2,..,P_n})\ \mathbf{P}$. This can also be placed into a linguistic from as $\Theta(\mathbf{P}_1,\mathbf{P}_2,\ldots,\mathbf{P}_n) \xrightarrow{F(\mathbf{P}_1,\mathbf{P}_2,\ldots,\mathbf{P}_n)} \mathbf{P}$. W Since there are potentially so many different types of interactions, a set of generic operators are used to indicate the presence of an interaction with the specifics to be spelled out if known. Thus there are

1. Free sequential (free interactive sum): $\mathbf{P}_1 \hat{\boxplus} \mathbf{P}_2$

2. Exclusive sequential (exclusive interactive sum): $\mathbf{P}_1 \boxplus \mathbf{P}_2$

3. Free concurrent (free interactive product): $\mathbf{P}_1 \hat{\boxtimes} \mathbf{P}_2$

4. Exclusive concurrent (exclusive product): $\mathbf{P}_1 \boxtimes \mathbf{P}_2$

Independence, sums and products are commutative, associative and distributive operations. Concatenation is non-commutative and non-associative in general. The zero process, **O**, is the process that does nothing.

An important and special form of interaction is the *coupling interaction*. Such interactions respect epistemological equivalence and thus are potentially reversible through a subsequent coupling interaction. An example is a rotation to a different eigenbasis as a result of an engagement with a measurement apparatus.

The impact of these different operations is best demonstrated using a *process graph*. The process graph $\mathcal{G}(\mathbf{P})$ of a process **P** is defined as follows: rounds 0 to N are laid out in order. At round 0 one places the informons of the prior causal tapestry. At round k, place each informon n that was generated during round k and draw a directed line from each prior informon in its content set G_n to n and label it with the causal distance between the two informons. Note that no lines link informons of the prior causal tapestry to one another or nascent informons to one another since no information passes among them. Let $\mathcal{G}(\mathbf{P})_n = \{n\} \cup \mathbb{G}_n$, the subgraph of $\mathcal{G}(\mathbf{P})$ consisting of n and its content set. The process graph is used to determine the causal manifold interpretation of the nascent causal tapestry and the global Hilbert space interpretation. If a process acts on the same prior causal tapestry it may produce a different process graph, thus a different history. The process covering map gathers together the global Hilbert space interpretations of these different process graphs, thus all of the possible histories required for a sum over histories calculation. A configuration space graph and configuration space covering map can be defined for products of processes.

The basic rules for applying these operations in combining processes are the following:

1. The free sum is only used for single systems and combining states which possess identical property sets (pure states).

2. The exclusive sum is used for single systems and combining states which possess distinct property sets (mixed states).

3. The free product is used for multiple systems which possess distinct character (scalar, spinorial, vectorial and so on) (for example coupling a boson and a fermion).

4. The exclusive product is used for multiple systems which possess the same character (for example coupling two bosons or two fermions).

References

1. Zayed AI. Advances in Shannon's sampling theory. Boca Raton: CRC Press. 1993.
2. Landau H. Necessary density conditions for sampling and interpolation of certain entire functions. Acta Mathematica. 1967; 117(1): 37-52.
3. Trofimova I. Sociability, diversity and compatibility in developing system: EVS approach. In: Formal Descriptions of Developing Systems. Nation J, Trofimova I, Rand J, Sulis W, editors. Dordrecht: Kluwer; 2002; p.231-248.
4. Borchers HJ; Sen RN. Mathematical Implications of Einstein-Weyl Causality. New York: Springer. 2006.
5. Maymon S, Oppenheim AV. Sinc interpolation of nonuniform samples. IEEE Trans Sig Processes. 2011; 59 (10): 4745-4758.
6. Conway, J.H. On numbers and games; A.K. Peters: Natick, NH, 2001.
7. Hodges W. Building models by games. New York: Dover Publications. 2006.
8. Hirsch R, Hodkinson I. Relation algebras by games. New York: Elsevier. 2002.
